# Physicochemical Properties, Cytocompatibility, and Biocompatibility of a Bioactive Glass Based Retrograde Filling Material

**DOI:** 10.3390/nano11071828

**Published:** 2021-07-14

**Authors:** Kazumasa Murata, Ayako Washio, Takahiko Morotomi, Thira Rojasawasthien, Shoichiro Kokabu, Chiaki Kitamura

**Affiliations:** 1 Division of Endodontics and Restorative Dentistry, Department of Oral Functions, Kyushu Dental University, Kitakyushu 803-8580, Japan; r17murata@fa.kyu-dent.ac.jp (K.M.); r13morotomi@fa.kyu-dent.ac.jp (T.M.); r06kitamura@fa.kyu-dent.ac.jp (C.K.); 2 Division of Molecular Signaling and Biochemistry, Department of Health Improvement, Kyushu Dental University, Kitakyushu 803-8580, Japan; r19Rojasawasthien@fa.kyu-dent.ac.jp (T.R.); r14kokabu@fa.kyu-dent.ac.jp (S.K.)

**Keywords:** bioactive glass, biocompatibility, cementoblast, cytocompatibility, physicochemical property, retrograde filling material

## Abstract

The ideal retrograde filling material that is easy to handle, has good physicochemical properties, and is biocompatible has not yet been developed. The current study reports the development of a novel bioactive glass based powder for use as a retrograde filling material that is capable of altering the consistency and hardening rate of mixtures when mixed with existing bioactive glass based cement. Furthermore, its physicochemical properties, in vitro effects on human cementoblast-like cells, and in vivo effects on inflammatory responses were evaluated. The surface of the hardened cement showed the formation of hydroxyapatite-like precipitates and calcium and silicate ions were eluted from the cement when the pH level was stabilized at 10.5. Additionally, the cement was found to be insoluble and exhibited favorable handling properties. No adverse effects on viability, proliferation, and expression of differentiated markers were observed in the in vitro experiment, and the cement was capable of inducing calcium deposition in the cells. Moreover, the cement demonstrated a lower number of infiltrated inflammatory cells compared to the other materials used in the in vivo mouse subcutaneous implantation experiment. These findings suggest that the retrograde filling material composed of bioactive glass and the novel bioactive glass based powder exhibits favorable physicochemical properties, cytocompatibility, and biocompatibility.

## 1. Introduction

Retrograde filling materials are required to hermetically seal the apical canal space and prevent bacteria or their toxins from entering the periapical tissue via the root canal system [1,2]. Because the material is in close proximity to the periapical tissue, the tissue response to the material is critical and may influence the outcome of surgical endodontic treatments. Due to the excellent sealing properties of super EBA (EBA), which was developed in the 1960s as a substitute for zinc oxide-eugenol cement, it has been used as a root-end filling material for several decades [3,4]. Although EBA exhibits a high strength owing to the presence of ethoxybenzoic acid, its setting times are unpredictable. Furthermore, tissue compatibility studies have demonstrated that EBA causes mild to moderate inflammatory reactions due to the presence of eugenol [5]. Numerous pivotal clinical results on the use of other filler materials such as mineral trioxide aggregate (MTA), in retrograde filling procedures, have been reported recently, and basic research to support its use has been conducted [6,7,8]. However, MTA has several clinical disadvantages, including a long setting time, high pH during setting, poor handling, and cytotoxicity in its freshly mixed state [9,10,11]. These disadvantages, therefore, demand the development of ideal retrograde filling materials.

Bioactive glass (BG), which is composed of calcium oxide, phosphorus pentoxide, silica, and sodium oxide, is one of the most studied biomaterials [12]. The clinical ability of BG to bond to bone is due to the formation of a bioactive layer of hydroxyapatite (HAp) on the glass surface without the need for surrounding fibrous tissue [13,14]. Recently, biomaterials based on BG fiber and nanoparticles have been recognized as valuable materials in the medical field due to their high surface area and versatility [15,16]. As a result, they have been used in drug delivery, tissue engineering, and dentistry [17,18,19,20]. Furthermore, the use of BG compounds in dental treatment is expected to increase [19,20].

Our previous study reports the development of a BG-based cement that demonstrated HAp precipitation on hardened surface, a stable pH value, cytocompatibility, and biocompatibility [21]. Recently, BG-based root canal sealer, which consists of two pastes with favorable properties of the prototype, has been available (Nishika Canal Sealer BG; CS-BG) [22], and has indicated favorable properties in root canal obturation [23]. The favorable physicochemical properties, biocompatibility, and clinical research of CS-BG support its use as a retrograde filling material. To further extend the desirable properties of CS-BG to retrograde filling materials, we developed a novel BG-based powder (code number: NSY-224) which can alter the consistency and hardening rate of mixtures when mixed with CS-BG.

Physicochemical properties are important parameters for the evaluation of materials [24]. Additionally, the evaluation of cytocompatibility and biocompatibility is essential in determining the safety of using biomaterials for clinical applications [25]. Different types of cells are cultured using the biomaterials to be tested, in order to determine the corresponding positive or negative influences on cell viability. After proving good cytocompatibility, the biomaterials are subjected to biocompatibility tests, in which they are applied at the subcutaneous level in animal models and collected subsequently to determine the presence of local reaction phenomena or systemic reactions. In the present study, the physicochemical properties, cytocompatibility, and biocompatibility of a new BG-based cement, obtained by mixing NSY-224 with CS-BG at an arbitrary ratio, were evaluated and compared with those of existing retrograde filling materials.

## 2. Materials and Methods

### 2.1. Materials

NSY-224 is composed of calcium silicate glass (molar ratio of Ca/P = 5.6, mass ratio of Ca/P = 7.4) and calcium hydroxide. Reagent grade SiO_2_, Ca(PO_3_)_2_, and CaCO_3_ were combined and the mixture was melted at 1500 °C. After cooling and crushing the molten mass, a powdery calcium silicate glass was obtained. The components of the glass were analyzed using X-ray fluorescence (Supermini200; Rigaku, Tokyo, Japan) and the absence of diffraction peaks were confirmed using X-ray diffraction (SmartLab 9 kW; Rigaku, Tokyo, Japan; XRD). CS-BG was obtained from Nippon Shika Yakuhin, Japan, White ProRoot MTA (Dentsply Tulsa Dental, Philadelphia, PA, USA; WMTA) and SuperEBA (Bosworth, Chicago, IL, USA; EBA) were used for comparison. The composition of each material is listed in Table 1.

### 2.2. Preparation of Cement Specimens

NSY-224 and CS-BG were used to fabricate the BG-based cement specimens. Specifically, NSY-224 was mixed with CS-BG at a weight of 0%, 20%, 40%, and 60% of the weight of CS-BG, denoted as NSY-224(0), NSY-224(20), NSY-224(40), and NSY-224(60), respectively. NSY-224(0) was obtained from CS-BG. WMTA and EBA were prepared according to the instructions of the manufacturers.

### 2.3. Field Emission Scanning Electron Microscope Analysis

The cement specimens were placed between two polypropylene films and incubated at 37 °C and a relative humidity of 100% for 24 h. The specimen size was approximately 5 mm, with a height of approximately 1 mm. After 4 days of immersion in simulated body fluid (SBF) at 37 °C, the specimens were washed with purified water. They were then dried for a single day in a desiccator. The surface structures of the platinum-coated specimens were analyzed using a field-emission scanning electron microscope (JSM-7000 F; JEOL, Tokyo, Japan; FE-SEM) operating at 15 kV.

### 2.4. X-ray Diffraction Analysis

The specimens were immersed in SBF for 4 days at 37 °C following which they were washed with purified water. They were then dried for a single day in a desiccator. The crystal structures of the specimens were subsequently analyzed using XRD. The X-ray beam angle 2θ (degree) range was set between 25°and 40°, and the specimen was examined using a scanning rate of 1°/min. The Cu X-ray source was operated at an electron beam current of 200 mA and an acceleration voltage of 45 kV. The patterns were identified using the SmartLab StudioⅡpowder XRD plugin (Rigaku, Tokyo, Japan).

### 2.5. pH Measurement

Immediately after mixing, 50 µL of the cement specimens were immersed in 10 mL of purified water for 0, 2, 4, 6, 8, 24, and 48 h. At each of the aforementioned time points, the pH of the liquid was measured using a pH meter (pH/ion meter F-73; HORIBA, Kyoto, Japan).

### 2.6. Inductively Coupled Plasma Optical Emission Spectrometry (ICP-OES)

Immediately after mixing, the cement specimens were immersed in purified water. After 1, 2, and 3 days of immersion, the supernatant was collected and filtered to remove cement particles using a filter with a pore size of 0.2 µm. The filtrates were subsequently used as sample solutions. Each sample solution was diluted by a factor of 100 using nitric acid (0.5 M) and quantitatively assessed using ICP-OES (Optima 8300; Perkin Elmer, Waltham, MA, USA).

### 2.7. Flow, Setting Time, Solubility and Disintegration, and Radio-Opacity

The test conditions for flow were modified in in reference to International Organization for Standardization (ISO) 6876: 2012 to compare the properties of each cement. Specifically, a weight of 4.8 kg was placed 1 min after the start of mixing, and the maximum and minimum diameters were measured 10 min after the start of mixing, and the average value was recorded. The setting time of each cement specimen was determined according to ISO 3107: 2011 for non-water-based zinc oxide/eugenol cements used in restorative dentistry for temporary restorations, and the solubility and radio-opacity of each cement specimen were determined according to ISO 6876: 2012 for root canal sealing materials. For each material, the tests were performed three times.

### 2.8. Culture of Human Cementoblast-Like Cells

An immortalized cell line of human cementoblast-like cells (HCEMs) was used. The HCEMs were kindly provided by Dr. Takashi Takata, and their characteristics have been previously described [27]. The HCEMs were routinely cultured in alpha-modification of Eagle’s medium (FUJIFILM Wako Pure Chemical, Osaka, Japan; MEMα) at 37 °C, in a humidified atmosphere containing 5% CO_2_. The media was supplemented with 10% (*v*/*v*) heat-inactivated fetal bovine serum (Sigma-Aldrich, Saint Louis, MO, USA; FBS), 100 units/mL penicillin (FUJIFILM Wako Pure Chemical, Osaka, Japan), and 100 µg/mL streptomycin (FUJIFILM Wako Pure Chemical, Osaka, Japan).

### 2.9. Cellular Morphological Analysis and Viable Cell Count

The effects of the materials on the HCEMs were examined using a 24-well cell culture insert system (Corning, NY, USA). The HCEMs were seeded at a density of 1 × 10⁴ cells/well and cultured in MEMα supplemented with 10% FBS. After 12 h, the medium was changed to MEM containing 1% FBS, and each cement specimen was placed on 8.0 µm pore size Transwell filter inserts. After 24 h, 48 h, and 72 h, the cells were observed using phase-contrast microscopy (CKX41; Olympus, Tokyo, Japan). They were then detached using 0.5% Trypsin-EDTA solution (Gibco, Grand Island, NY, USA). Subsequently, equal volumes of cell suspension and 0.5% trypan blue staining solution (Nacalai Tesque, Kyoto, Japan) were loaded into the TC10 counting slide (BIO-RAD Laboratories, Hercules, CA, USA). Finally, the number of viable cells were counted using a TC10 automated cell counter (BIO-RAD Laboratories, Hercules, CA, USA).

### 2.10. Immunocytochemistry Analysis

After permeabilization and blocking with phosphate-buffered saline (PBS) containing 5% goat serum and 0.3% Triton X-100 for 20 min at room temperature, the HCEMs were incubated with a primary antibody overnight at 4 °C. Either the anti-Ki-67 rabbit polyclonal antibody (ab15580, Abcam, Cambridge, UK) or the anti-caspase-3 rabbit polyclonal antibody (#9664, Cell Signaling, Danvers, MA, USA) was used for the assay. The target proteins were visualized using an Alexa 546-conjugated secondary antibody (1:1000 dilution; Invitrogen, Carlsbad, CA, USA) and imaged with a microscope (ABZ-9000; Keyence, Tokyo, Japan). Furthermore, the cells were stained with DAPI (1:1000 dilution; Vector Laboratories, Burlingame, CA, USA) to visualize the cell nuclei. The numbers of DAPI-positive cells and Caspase-3-positive cells were counted, and the ratio of Caspase-3 positive cells to DAPI-positive cells was calculated. The counting was conducted in a double-blind fashion.

### 2.11. Gene Expression Experiments

The HCEMs were seeded at a density of 1 × 10⁵ cells/well and were cultured in MEMα containing 10% FBS. Twelve hours after seeding the cells, the medium was changed to MEMα containing 10% FBS, with or without an osteogenic supplement (OS). The primary constituents of the supplement included 50 µg/µL ascorbic acid (Sigma-Aldrich, Saint Louis, MO, USA), 10 mM β-glycerophosphate (Wako Pure Chemical, Osaka, Japan), and 50 nM dexamethasone (Sigma-Aldrich, Saint Louis, MO, USA). Each cement specimen was then placed on Transwell filter inserts with an 8.0 µm pore size. After 7 days, the total RNA was isolated from the cells using a FastGene TM RNA Basic Kit (Nippon Genetics, Tokyo, Japan), and the cDNA was synthesized using the High Capacity cDNA Reverse Transcription Kit (Applied Biosystems, Waltham, MA, USA). Subsequently, a SYBR-green-based quantitative reverse transcription polymerase chain reaction (qPCR) was performed in 96-well plates using PowerUp SYBR Green Master Mix (ThermoFisher Scientific, Waltham, MA, USA) in a QuantStudio 3 Real-Time PCR System (ThermoFisher Scientific, Waltham, MA, USA). The expression levels of individual genes were normalized to those of *β-ACTIN*, *TATA-box binding protein (TBP)*, and *hypoxanthine phosphoribosyltransferase 1 (HPRT1)* using the 2^−^^ΔΔCt^ method [28,29]. The primer pairs used for the evaluation of cementoblast differential marker genes are shown in Table 2.

### 2.12. Alkaline Phosphatase Activity

The HCEMs were seeded at a density of 1 × 10⁵ cells/well and cultured in MEMα containing 10% FBS. Twelve hours after seeding the cells, the culture medium was changed to an OS medium, and each cement specimen was placed on Transwell filter inserts with a pore size of 8.0 µm. The alkaline phosphatase (ALP) activity was measured after 7 days, using a *p*-nitrophenylphosphate assay kit (LabAssay ALP Kit; Wako Pure Chemical, Osaka, Japan). After 15 min of incubation at 37 °C, the absorbance of *p*-nitrophenylphosphate was determined using a microplate reader at 405 nm (Bio-Rad iMark TM; BIO-RAD Laboratories, Hercules, CA, USA), and the specific activity of ALP (U/µL) was calculated. Simultaneously, the cells were fixed with 4% paraformaldehyde (FUJIFILM Wako Pure Chemical, Osaka, Japan; PFA) in PBS for 10 min at room temperature and stained using a nitro-blue tetrazolium chloride/5-bromo-4-chloro-3′-indolylphosphate *p*-toluidine salt stock solution (Sigma-Aldrich, Saint Louis, MO, USA), based on the manufacturer’s instructions. The relative staining intensity was analyzed semi-quantitatively using ImageJ software (National Institutes of Health, Bethesda, MD, USA).

### 2.13. Detection of Extracellular Calcium Deposition

The HCEMs were seeded at a density of 1 × 10⁵ cells/well and cultured in MEMα containing 10% FBS. Twelve hours after seeding the cells, the medium was changed to an OS medium, and each cement specimen was placed on Transwell filter inserts with a pore size of 8.0 µm. After 28 days, the specimens were stained using Alizarin Red S (Sigma-Aldrich, Saint Louis, MO, USA), which assessed mineralization. In brief, the cells were fixed with 4% PFA for 10 min at room temperature, then washed thrice with PBS and stained with 1% alizarin red S solution. After washing with distilled water, phase-contrast microscopy was used to examine the stained area of each individual well. Subsequently, the stained calcium depositions were dissolved in 10% (*w*/*v*) cetylpyridinium chloride (Sigma-Aldrich, Saint Louis, MO, USA; CPC) solution on a rocking shaker. After 1 h, the eluted solutions were transferred to 96-well plates and measured at 562 nm using a microplate reader.

### 2.14. Subcutaneous Implantation and Histological Analysis

The animal experimentation was conducted in accordance with the guidelines for animal care at Kyushu Dental University. The ethical approval was obtained from the Institutional Panel for Animal Care (No. 19-024). The implantation was carried out on six-week-old male C57BL/6 mice under semi-barrier conditions. They were anaesthetized intraperitoneally with thiopental (40 mg/kg, Ravonal; Nipro ES Pharma, Osaka, Japan). Subsequently, two incisions were made on the dorsal region of each mouse. The skin, lateral to the incisions was pinched, and subcutaneous dissection was performed using blunt-ended scissors. Each animal received two sterile polyethylene tubes (10 mm in length and 1.5 mm in diameter) filled with each cement. Mice implanted with empty tubes were used as controls. The incisions were then closed using silk 4-0 sutures. After 1 week, the animals were euthanized by administering an anesthetic overdose with isoflurane (Wako Pure Chemical, Osaka, Japan). Biopsies of the skin and subcutaneous tissues (2 × 1 cm) containing the implants were obtained with 5 mm safety margins. The subcutaneous tissues containing the tubes were excised and fixed in 4% PFA/PBS overnight. Subsequently, the specimens were trimmed parallel to the tube, leaving at least 2 mm tissue on each side. They were then cut into two equal halves, and the tubes were removed. Following that, the specimens were immersed in serially increasing concentrations of ethyl alcohol for dehydration, cleared in xylene, and embedded in paraffin at 58–62 °C. The samples were trimmed parallel to the long axis of the tube to reveal the region of interest (tube opening). Subsequently, serial sections with a thickness of 6 µm were prepared for staining with hematoxylin and eosin stain (H and E). The histological examination was performed using a light microscope (BX50F; Olympus, Tokyo, Japan).

To perform immunohistochemical analysis, paraffin-embedded blocks were cut into 6 µm thick sections and the paraffin was removed from the tissue sections using Xylene. The slides were then rehydrated in an ethanol gradient via incubation for 3 min in 100%, 95%, 80%, 70%, and 50% ethanol solutions. They were then washed in deionized water and PBS for 5 min at room temperature. Subsequently, unspecific antibody binding was blocked by incubating the slides for 30 min at room temperature in PBS containing 5% goat serum. This solution was used to wash between the subsequent steps. To carry out CD11b or CD45 staining, the sections were incubated for 2 h at room temperature with Anti-CD11b rabbit monoclonal antibody (1:500 dilution; ab184308, Abcam, Cambridge, UK) or anti-CD45 rabbit monoclonal antibody (1:200; #70257, Cell Signaling, Danvers, MA, USA). The slides were then washed and incubated for 1 h at room temperature with an Alexa 546-conjugated secondary antibody (1:1000 dilution; A11035, Invitrogen, Carlsbad, CA, USA) and then imaged with an ABZ-9000 light microscope. To visualize the cell nuclei, the slides were stained with DAPI (ProLong^TM^ Diamond Antifade Mountant with DAPI, P36962, Invitrogen, Carlsbad, CA, USA).

### 2.15. Statistical Analysis

All experiments were repeated three times to ensure that the results were reproducible. Statistically significant differences were determined using one-way analysis of variance (ANOVA) combined with Tukey’s test. R software (Version R 3.6.3, The R Foundation, Vienna, Austria) was used for the analysis. The data are expressed as mean ± SD. Furthermore, *p*-values lower than 0.05 and 0.01 were regarded as significant (* *p* < 0.05, ** *p* < 0.01, *** *p* < 0.001).

## 3. Results

### 3.1. Surface Structures of Cements

**Figure 1:**
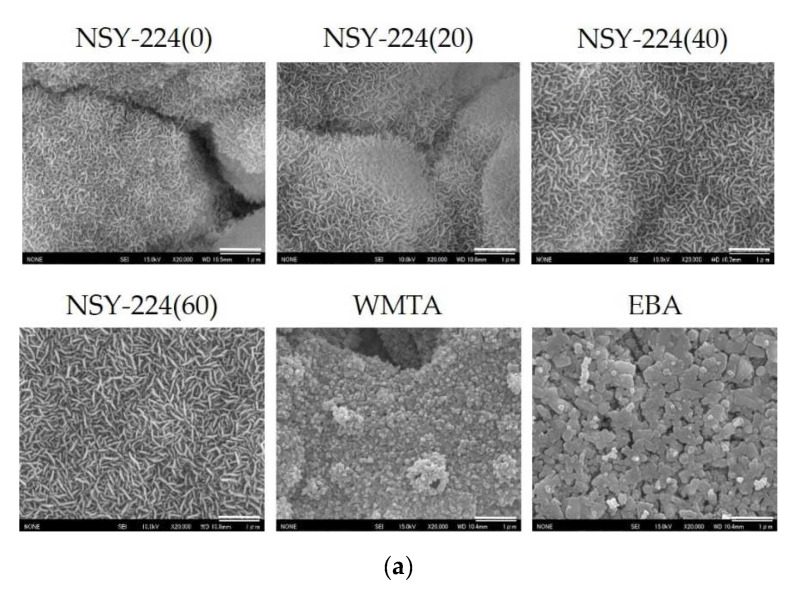

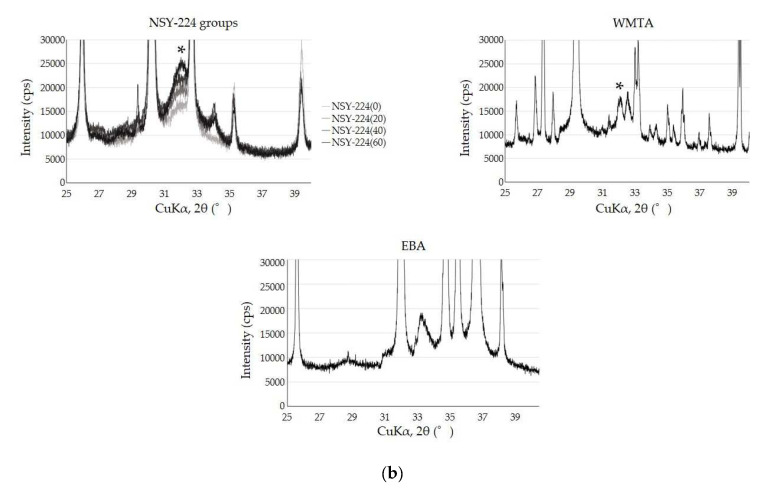
Formation of apatite and crystal analysis. (**a**) FE-SEM images of cement surface. Scale bars, 1 µm. (**b**) Crystal analysis using X-ray diffraction. * in the figure shows the position of the apatite peak.

Figure 1 shows the surface structures of the NSY-224 group, WMTA, and EBA specimens. After 4 days of immersion in SBF, typical spherule structures of petal-like precipitates were observed on the surfaces of the NSY-224 group and WMTA specimens. Similar structures were absent on the surface of the EBA specimen (Figure 1a). Evaluation of the cement surface crystalline showed that the petal-like crystals were composed of HAp. The crystallinity tended to increase as the combination ratio of NSY-224 increased (Figure 1b).

### 3.2. pH Changes and Ionic Elution of Cements

**Figure 2:**
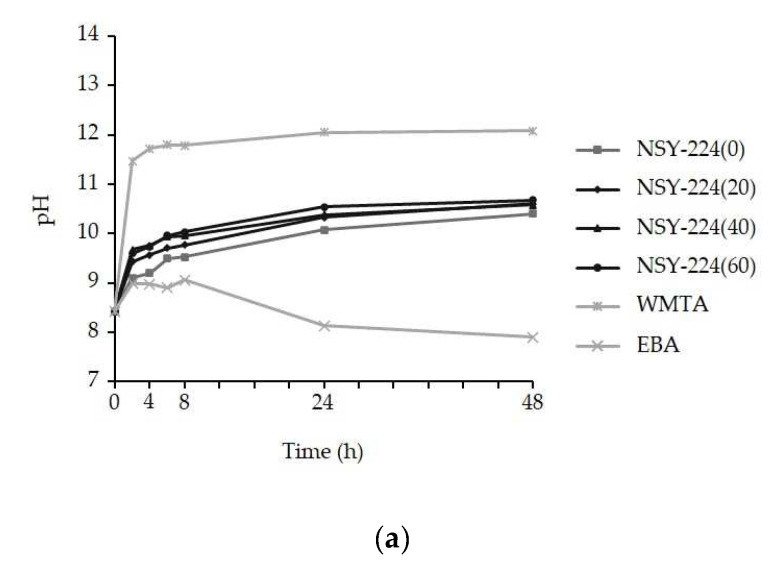

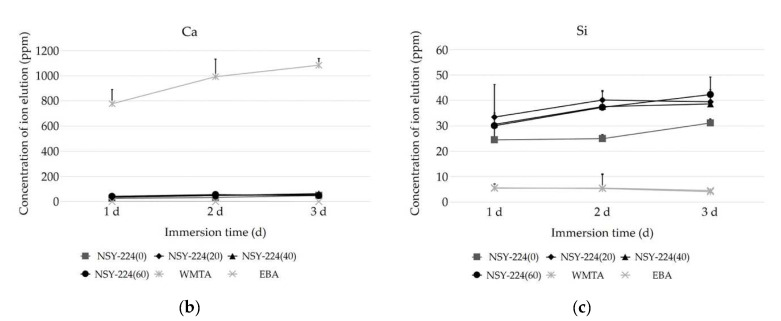
pH changes and ion elution of each cement specimen. (**a**) pH changes of the cement specimens immersed purified water. (**b**) Calcium ion and (**c**) silicon ion eluted from each cement. The data are expressed as means ± SDs (*n* = 3).

Figure 2 shows the pH changes and the ion elution of the cement specimens immersed purified water for various periods. In all the cement specimens, an intense increase in pH was observed during the 2 h period. During the 48 h period, the pH of the NSY-224 group, WMTA, and EBA stabilized at 10.5, 12.1, and 7.9, respectively (Figure 2a). In the ionic elution, there were no differences in the dissolution rate between the cement specimens containing NSY-224 added at various combination ratios. The concentration of eluted calcium ions was the highest for WMTA (Figure 2b). The concentration of eluted silicon ions was higher for the NSY-224 group than for WMTA (Figure 2c). There was less ion elution in the case of EBA (Figure 2b,c).

### 3.3. ISO Evaluation

Table 3 shows the results of flowability, setting time, solubility and disintegration, and radio-opacity obtained via the ISO evaluation. The flowability of NSY-224(0), (20), (40), (60), WMTA, and EBA was 33.1 mm, 29.7 mm, 22.8 mm, 16.5 mm, 10.6 mm, and 33.1 mm, respectively. The setting time of NSY-224(0), (20), (40), (60), WMTA, and EBA was 150 min, 80 min, 40 min, 8 min, 10 min, and 1 min, respectively. The solubilities of NSY-224(0), (20), (40), and (60) were either 0.5% or 0.6%. Further, the solubilities of the WMTA and EBA specimens were 2.4% and 0.1%, respectively. None of the cement specimens showed disintegration. The radio-opacity of NSY-224(0), (20), (40), and NSY-224(60) was 6 mm Al, 5 mm Al, 5 mm Al, and 4 mm Al, respectively, and that of WMTA and EBA was 5 mm Al.

### 3.4. Effects of Cements on Morphology, Viability, Proliferation, and Apoptosis of HCEMs

**Figure 3:**
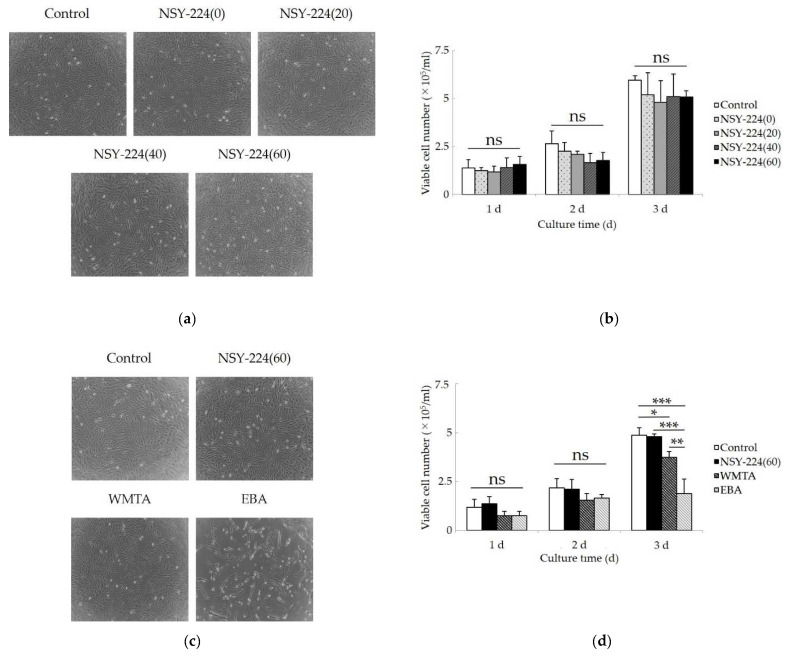

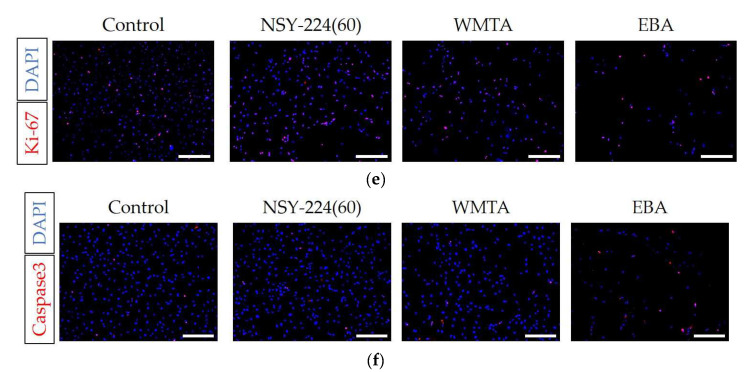
Effects of cements on morphology, viability, proliferation, and apoptosis of HCEMs. (**a**,**c**) Phase-contrast images of HCEMs cultured with each cement for 72 h. (**b**) Effect of mixing ratio of NSY-224 on the number of viable cells. (**d**) Effect of various materials on the number of viable cells. (**e**,**f**) Fluorescent images of Ki-67 (**e**) or Caspase3 (**f**) positive immunostaining in cells cultured with each cement for 60 h. Scale bars, 500 µm. The data are expressed as means ± SDs (*n* = 3); ns: not significant; * *p* < 0.05, ** *p* < 0.01, *** *p* < 0.001.

Figure 3 shows the morphology, viability, cell proliferation, and apoptosis of HCEMs located under the cement specimens in the filter inserts. Under normal conditions without cement specimens, the cells were elongated and spindle-shaped. Neither the NSY-224 group nor WMTA affected the morphology of the cells compared to that under normal conditions (Figure 3a,c). Exposure to EBA induced vacuoles in the cells (Figure 3c). In the count assay of viable cells, no significant difference in the number of viable cells compared to that in normal conditions was observed for the NSY-224 group (Figure 3b), and the number of viable cells in the case of WMTA was slightly lower than that for NSY-224(60). Additionally, the number of viable cells in the case of EBA was substantially lower than that of the other specimens (Figure 3d). The immunocytochemistry assay confirmed that the numbers of both Ki-67-positive cells and caspase-3-positive cells in the cases of NSY-224(60) and WMTA were not different from that for the control, although the number of caspase-3-positive cells was the highest for EBA among the four groups (Figure 3e,f). A semiquantitative analysis revealed that the ratio of the numbers of caspase-3-positive cells to DAPI-positive cells for the control, NSY-224(60), WMTA, and EBA were 12%, 13%, 15%, and 74%, respectively.

### 3.5. Effects of Cements on Cementoblastic Differentiation and Calcification of HCEMs

**Figure 4:**
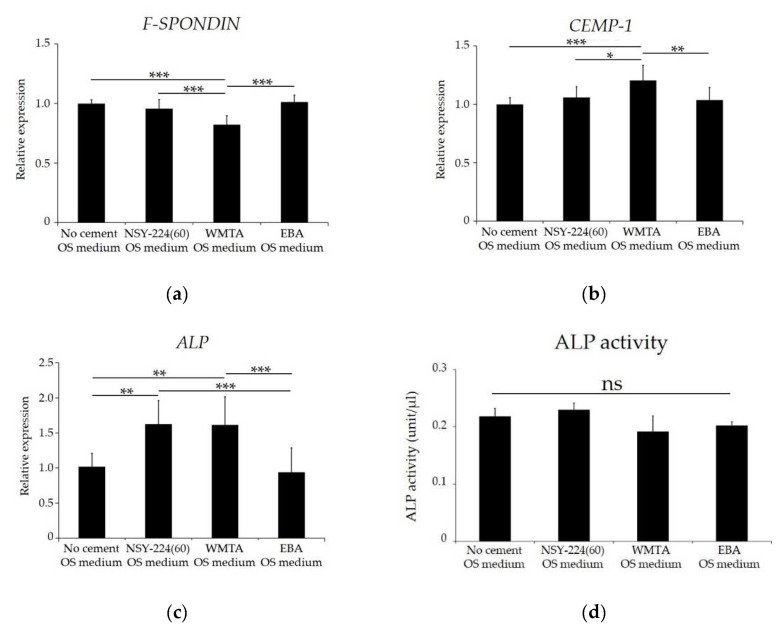

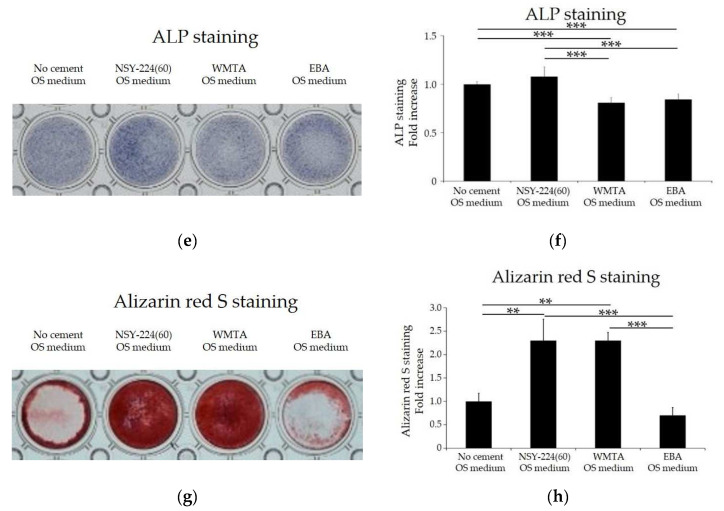
Effects of cements on differentiation and mineralization of HCEMs. (**a**–**c**) HCEMs were cultured with each cement in OS medium for 3 days, and mRNA levels of the indicated genes were determined by qPCR. ALP activity (**d**), ALP staining (**e**), semiquantitative analysis of the ALP staining using Image J (**f**), alizarin red S staining (**g**), and quantitative analysis of alizarin red S staining using CPC solution (**h**) of HCEMs cultured with each cement in OS medium. The data are expressed as means ± SDs (*n* = 3); ns: not significant; * *p* < 0.05, ** *p* < 0.01, *** *p* < 0.001.

Figure 4 shows the effects of each cement on the cementoblast differential marker genes, ALP activity, and the calcium deposition of HCEMs. The mRNA levels of *CEMP-1* and *F-SPONDIN* were not significantly affected by NSY-224(60) and EBA when compared to the OS medium without cement specimens. In WMTA, the mRNA expression of *F-SPONDIN* was significantly decreased (Figure 4a), while that of *CEMP-1* was significantly increased (Figure 4b) compared to the other groups. Although NSY-224(60) and WMTA significantly increased ALP mRNA levels (Figure 4c), the three cement groups and the OS medium without cement specimens had no significant effect on ALP activity (Figure 4d). On the other hand, the results of ALP staining showed that the staining intensities of NSY-224(60) were significantly higher than those of WMTA and EBA (Figure 4e,f). Furthermore, Alizarin red S staining revealed that the staining intensities of NSY-224(60) and WMTA were higher than those of the OS medium without cement specimens, while the staining intensity of EBA was lower than those of NSY-224(60) and WMTA (Figure 4g,h).

### 3.6. Effects of Cements on Inflammatory Responses In Vivo

**Figure 5:**
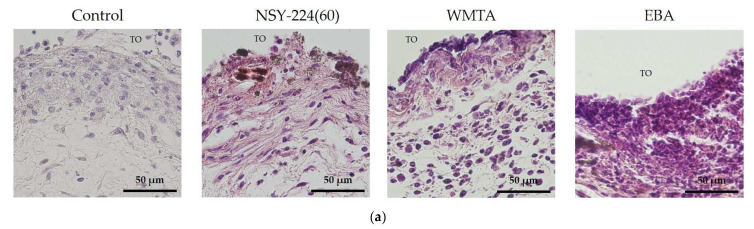

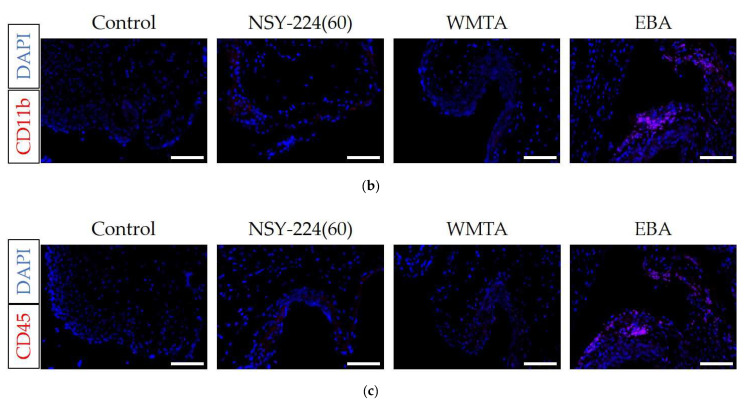
Effects of cements on inflammation in vivo. Histologic findings corresponding to mouse subcutaneous tissue adjacent to each cement specimen on the 7th day. Sections were stained with hematoxylin–eosin (**a**) and observed using light microscopy (×400 magnification). Scale bars, 50 µm. TO: tube opening. (**b**,**c**) Fluorescent images of the myeloid cell marker CD11b (**b**) and leukocyte marker CD45 (**c**). Sections were incubated with Alexa 546 fluorescent secondary antibodies and mounted with mounting media containing the nuclei stain DAPI. Red correlates with the neutrophil and monocyte marker and blue staining correlates with the DAPI. Scale bars, 100 µm (×40 magnification).

Figure 5 shows the histopathological findings of the subcutaneous implantation study. On the 7th postoperative day, several inflammatory cell infiltrations were observed in the three cement groups. However, NSY-224(60) and WMTA exhibited lower inflammatory cell infiltration than EBA. Particularly, EBA tended to accumulate more inflammatory cells than the others. In addition, the formation of fibrous capsules was evident in most of the specimens (Figure 5a). The results of immunohistochemistry, therefore, showed that EBA group had a greater infiltration of CD11b and CD45 positive cells than the other groups (Figure 5b,c). Without the primary antibody (negative control), the sections exhibited no immunopositive cells (data not shown).

## 4. Discussion

When BG is combined with phosphate ions, a calcium-phosphate-rich layer forms that adhere to dentin [30], offering good abrasion resistance and acceptable mechanical properties [31]. The purpose of this study was to develop a new BG-based retrograde filling material and to analyze its physicochemical properties. This was carried out by mixing NSY-224 (which contains BG powder) and CS-BG (a commercially available root canal sealer). After immersion in SBF, FE-SEM imaging and XRD patterns revealed typical spherules of petal-like HAp crystals, which were deposited on the surfaces of the NSY-224-group specimens. This appears to be a characteristic shared by a wide variety of biomaterials, including BG [32]. Additionally, immersing WMTA in SBF causes HAp to precipitate onto the surface in the form of needle-like crystals [33]. Conversely, it was suggested that the crystals are not formed on the surfaces of the EBA specimens because EBA does not contain bioactive ceramics. Moreover, it was also reported that apatite deposits within collagen fibers and the interfacial layer composed of apatite is accompanied by tag-like structures that extend into the dentinal tubules [34]. Thus, the apatite layer of NSY-224-group specimens and WMTA may play a role in triggering dentinogenic activity, thereby minimizing leakage by filling the gap along the interfaces. Furthermore, they may also interact with dentin via intra-fiber apatite depositions, to promote mineral nucleation on dentin. Additionally, it is well established that HAp is least soluble at pH values 8–9, as determined by the solubility curve of HAp with respect to pH. Therefore, it is suggested that HAp crystallization is more difficult on NSY-224-group specimens than on WMTA, resulting in stable conditions. Furthermore, the difference in calcium ions release of 20–30 times is equivalent to a pH difference of 1.3–1.5. Because CaO-SiO_2_, a major component of WMTA, has a higher solubility than CaO-SiO_2_-P_2_O_5_, which comprises a major component of NSY-224 and CS-BG, Ca ion release from WMTA is comparatively higher than other cement. Thus, the pH of NSY-224 groups and WMTA is suggested to be caused by calcium ions eluted from those materials.

A suitable retrograde filling material must form an apical seal that prevents the irritant from the root canal system from leaking into the periapical tissue [35,36]. Good handling and insolubility are desirable properties for dental materials; moreover, they are ideal for retrograde filling materials [37]. In the present study, NSY-224(20), (40), and (60) had shorter setting times than NSY-224(0). In particular, the setting time of NSY-224(60) was much shorter than that of WMTA, suggested that NSY-224(60) exhibits more favorable setting time than WMTA. Furthermore, EBA exhibited the shortest setting time in the experimental group. NSY-224(20), (40), and (60) have lower flow rates than NSY-224(0) and EBA. The flow rate of WMTA was the shortest in the experimental group. These results suggest that NSY-224(60) and WMTA possess good handling characteristics as retrograde filling materials. Nevertheless, the solubility of NSY-224 groups and EBA was lower than that of WMTA, suggesting that NSY-224(60) and EBA have poor solubility. Furthermore, the radio-opacity of the NSY-224-group specimens decreased with increasing NSY-224 combination ratio but remained comparable to that of MTA and EBA. As a result, NSY-224(60) is suggested to be the most suitable retrograde filling material in the experimental group.

Endodontic biomaterials frequently come in direct contact with periodontal tissues. As a result, they must be cytocompatible and biocompatible with host tissues. The cytocompatibility of retrograde filling materials has been evaluated based using a variety of cell lines, including osteoblasts [38], gingival fibroblasts [39], periodontal ligament cells [40], and keratinocytes [41]. On the contrary, the effect of such materials on cementoblasts or cementoblast-like cells has not been investigated, although cementoblasts play important roles in the repair and maintenance of cementum. In this regard, we used HCEMs, which are human cementoblast-like cell lines [27], because the evaluation of the cytocompatibility of materials with cementoblast-like cells is beneficial for clinical applications. Biological tests with inadequate material conditions, such as those related to setting and sterilization, have been conducted in many studies although a freshly mixed material is applied to the root canal in clinical practice and elution from this material affects surrounding periapical tissue [42,43]. As a result, we assessed cytocompatibility and biocompatibility using freshly mixed materials, ensuring that our method is more representative of clinical conditions.

The cytocompatibility of the new BG cement was evaluated by examining the changes in the morphology, viability, proliferation, apoptosis, and differentiation of HCEMs. Our data showed that NSY-224(60) did not adversely affect cell viability. This is attributed to the suitable pH and calcium ions levels in NSY-224(60). It has been reported that calcium ions stabilize or enhance the viability of dental pulp cells [44], periodontal ligament cells [45], and odontoblasts [46]. Additionally, pH is also an important factor that influences the wound healing process by changing throughout the process [47,48,49]. In our previous study, we revealed that periodontal ligament cells, and osteoblast-like cells cultured with the prototype of CS-BG within the pH range of 9 to 10 had no adverse effects [21]. In the other previous study, when fibroblasts were cultured within the pH range 6.5–10.5, the cells tolerated the pH range very well and no major differences in viability were observed. In addition, the cells decreased in a time-dependent manner when cultured at pH > 11.5 [49]. The pH of NSY-224(60) and WMTA was approximately 10.5 and 12, respectively. It is suggested that calcium ions eluted from NSY-224-group specimens and their pH correspondingly influence the viability of cementoblast-like cells. By contrast, the viability of HCEMs cultured with WMTA was significantly lower than that of the control, which was considered to be caused by the high pH after fresh mixing. In the present study, EBA had the most cytotoxic effect although the pH of EBA was nearly neutral. Several studies have reported that the high cytotoxicity of EBA is due to the free eugenol contained in it [45,50]. Therefore, in this case, the eugenol in EBA may have also had a cytotoxic effect on HCEMs without affecting the pH.

Because the Ki-67 nuclear antigen does not recognize cells in the G0 phase, it is associated with cell proliferation [51,52]. Apoptosis rose to prominence as a central concept in the study of numerous biological processes. Among the enzymes and factors involved in apoptosis, caspase-3 degrades a variety of cytoplasmic and nuclear proteins and activates nucleases, which promote DNA degradation [53]. Thus, the detection of caspase-3 by immunocytochemistry assays has been used to identify and confirm cell death via apoptosis [54]. Since NSY-224(60) and WMTA did not change the number of Ki-67- and caspase-3-positive cells, both materials are considered to have no apoptotic effect and induce cell proliferation. By contrast, EBA strongly increased the number of caspase-3-positive cells. Based on these results, we can explain the cytotoxicity of EBA as follows: free eugenol in EBA induced apoptosis and reduced the number of viable cells at the proliferation stage, as opposed to the differentiation stage, because EBA had little influence on the differentiation of HCEMs in our experiments.

Ions released from BG and ceramics, such as calcium and silicate ions, have been reported to influence osteogenic differentiation and calcification [55,56]. Additionally, cell responses to ions depend on the ion type and combination [56,57,58]. Previous studies have reported that calcium ions released from MTA increased the odontogenic differentiation and calcification of various types of cells such as odontoblasts, periodontal ligament cells, and cementoblasts [59,60,61]. However, the effects of retrograde filling materials on the cementogenic differentiation of cementoblasts and cementoblast-like cells have not been reported to date. We focused on cementoblast differentiation related genes (*F-SPONDIN* and *CEMP-1*) [62,63,64] and an early marker of osteogenic and cementogenic differentiation (*ALP*) in the present study. While, F-spondin is important for inducing cementoblastic differentiation [63], CEMP-1 is necessary to decide the cementoblastic phenotype [62]. Since in our data, NSY-224(60) and EBA had no adverse effect on the cementogenic differentiation related gene expression of HCEMs. However, WMTA decreased *F-SPONDIN* but stimulated the expression of *CEMP-1* of HCEMs, suggesting that WMTA may regulate the fate commitment of cementoblasts. On the other hand, NSY-224(60) increased the gene expression and the activity of ALP, while WMTA reduce the activity of ALP, on the cementogenic differentiation of HCEMs. However, NSY-224(60) and WMTA increased calcification (calcium deposition), which is presumed to be caused by calcium ions released from NSY-224(60) and WMTA, considering that extracellular calcium ions are important factors that contribute to calcification [65,66]. Therefore, it is suggested that retrograde filling materials composed of calcium and silicate maintain cementogenic differentiation and increase the calcification of cementum, and the hard tissue (cementum) may be formed adjacent to these materials and on the root surface after performing retrograde filling of the root canal via apicoectomy.

Numerous studies have been conducted to evaluate the subcutaneous inflammatory reactions to retrograde filling materials [67,68]. The materials are placed in tubes to prevent their diffusion into the connective tissue, simulating the root canal [69]. In the present study, we focused on CD11b and CD45. CD11b, an α chain of the leukocyte β2-integrin, is a representative marker of the myeloid cells, such as those with monocyte and granulocyte lineage [70]. Cd45 is a type 1 transmembrane protein tyrosine phosphatase, which is expressed by all the hematopoietic stem cells except that of erythrocytes and platelets [71]. Our results showed a milder tissue reaction to NSY-224(60) and WMTA than to EBA. Therefore, it is suggested that NSY-224(60) produces a similar or less inflammatory reaction than existing retrograde filling materials.

## 5. Conclusions

We demonstrated that the new BG-based retrograde filling material developed in this study possessed comparable or superior physicochemical properties, cytocompatibility, and biocompatibility to existing retrograde filling materials and was capable of overcoming WMTA’s disadvantages.

## Figures and Tables

**Table 1:** Components of NSY-224, CS-BG, WMTA, and EBA.

Materials	Code	Compositions *
Newly developed BG powder	NSY-224	Calcium silicate glass and calcium hydroxide
Nishika Canal Sealer BG	CS-BG	Paste A: bismuth subcarbonate, fatty acid, and silicon dioxide
		Paste B: calcium silicate glass, magnesium oxide, purified water, silicon dioxide, and others
White ProRoot MTA	WMTA	Powder: tricalcium silicate, dicalcium silicate, tricalcium aluminate, bismuth oxide, and calcium sulfate
		Liquid: sterile water
SuperEBA	EBA	Powder: zinc oxide, alumina, and natural resin
		Liquid: ortho-ethoxy benzoic acid and eugenol

* The composition of CS-BG was based on the available information included in the product information. The composition of WMTA was inferred from a previous study [26]. The composition of EBA was obtained based on the instructions for use.

**Table 2:** The primers for real-time PCR.

Target Gene		Primer Sequence (5′ to 3′)
*CEMP-1*	Forward:	ACACTGGTGCCTCCCATACT
(NM_001048212)	Reverse:	TCAATAACCCTATCTCTTCACACATC
*F-SPONDIN*	Forward:	TGTCGATGATATTGTAGCTGACC
(NM_006108)	Reverse:	CAGGTTTCAGGGGTGTCATC
*ALP*	Forward:	CCGGATGTTACCGAGAGC
(X_55958)	Reverse:	GTGGGTCTCTCCGTCCAG
*β-ACTIN*	Forward:	CCAACCGCGAGAAGATGA
(NM_001101)	Reverse:	CCAGAGGCGTACAGGGATAG
*TBP*	Forward:	GAACATCATGGATCAGAACAACA
(NM_003194)	Reverse:	ATAGGGATTCCGGGAGTCAT
*HPRT1*	Forward:	TGACCTTGATTTATTTTGCATACC
(NM_000194)	Reverse:	CGAGCAAGACGTTCAGTCCT

**Table 3:** Evaluation of the cement specimens based on ISO method.

Specimen	NSY-224(0)	NSY-224(20)	NSY-224(40)	NSY-224(60)	WMTA	EBA
Flow	33.1 mm	29.7 mm	22.8 mm	16.5 mm	10.6 mm	33.1 mm
Setting time	150 min	80 min	40 min	8 min	10 min	1 min
Solubility	0.6% No disintegration	0.5% No disintegration	0.6% No disintegration	0.5% No disintegration	2.4% No disintegration	0.1% No disintegration
Radio-opacity	6 mm Al	5 mm Al	5 mm Al	4 mm Al	5 mm Al	5 mm Al

## Data Availability

The data that support the findings of this study are available from the corresponding author, Ayako Washio, upon reasonable request.
